# Optimal fertilizer rate and sowing density can improve oat quality, yield and N and P comprehensive efficiency in the Loess Plateau of China

**DOI:** 10.3389/fpls.2025.1604427

**Published:** 2025-06-13

**Authors:** Ruochen Zhang, Yue Wang, Kamran Malik, Jianjun Wang, Guiqin Zhao, Chunjie Li

**Affiliations:** ^1^ State Key Laboratory of Herbage Improvement and Grassland Agro-ecosystems, Lanzhou University, Lanzhou, China; ^2^ Key Laboratory of Grassland Livestock Industry Innovation, Ministry of Agriculture and Rural Affairs, Beijing, China; ^3^ Engineering Research Center of Grassland Industry, Ministry of Education, Beijing, China; ^4^ Gansu Tech Innovation Center of Western China Grassland Industry, Lanzhou, China; ^5^ College of Pastoral Agriculture Science and Technology, Lanzhou University, Lanzhou, China; ^6^ Pratacultural College, Gansu Agricultural University, Lanzhou, China

**Keywords:** oat, sowing density, fertilizer rate, yield, quality, nutrients use efficiency

## Abstract

**Introduction:**

Oat is a nutritious native species in Loess Plateau for forage and animal husbandry. By focusing on the sowing density and fertilizer rate, oats can achieve better productivity performance while maximizing nutrients use efficiency. However, the information on the responses of oats yield, quality and nutrients use efficiency to fertilization and sowing density is limited.

**Methods:**

In this study, from 2022 to 2023, a two-factor randomized block design field trial was conducted in Loess Plateau. The three sowing densities (SD, L: 75 kg/ha; M: 150 kg/ha; H: 225 kg/ha) were coupled with six fertilizer rates (FR, N0P0: no fertilization; N2: 100 N kg/ha; P2: 90 P kg/ha; N1P2: 50 N kg/ha, 90 P kg/ha; N2P1: 100 N kg/ha, 45 P kg/ha; N2P2: 100 N kg/ha, 90 P kg/ha). The effects of FR and SD on oat forage yield, quality, agronomic efficiency (AE), N/P content, uptake and its uptake efficiency and recover efficiency were investigated. In order to compare N/P efficiency more intuitively, we combined N/P content, N/P uptake, N/P uptake efficiency and N/P recover efficiency to calculate N/P comprehensive efficiency.

**Results:**

Overall, the M-N1P2 treatment promoted the oat growth and achieved the maximum oat forage yield and quality. In the M treatment, the average crude protein (CP) content, relative feed value (RFV), forage yield, CP yield, N content, P content, N uptake and P uptake increased by 20.2%, 4.9%, 73.2%, 100%, 30.4%, 26.3%, 128.5% and 118.4%, respectively, compared with those under the no fertilization treatment; while the average neutral detergent fiber (NDF) decreased by 2.6% in the N1P2 treatment compared to no fertilization treatment. The optimum agronomic efficiency (AE), N uptake efficiency and N recover efficiency were also observed under M-N1P2 during both years. The comprehensive analysis revealed M-N1P2 also had the highest N comprehensive efficiency and P comprehensive efficiency among all treatments. The results of correlation analysis revealed significant positive correlations (*P<* 0.05) of forage yield and CP with nutrients efficiency of oat, but negative correlations with fiber content.

**Discussion:**

This study determined the appropriate sowing density and fertilization rate (M-N1P2) for cultivating oat in Loess Plateau and provided a foundation for promoting productivity of oats.

## 1 Introduction

Oat has high yield and nutritional quality, and is suitable for cultivation over a wide range of soil types and climatic conditions (Jakubus and Graczyk, 2022). It is the most widely grown annual forage cereal in the world (Andrzejewska et al., 2019). Due to oat forage’s rich composition of nutrients, including proteins, antioxidants, and various amino acids, it possesses a high nutritional value. There is growing interest in the oat cultivation as a result of an increasing appreciation of the feeding benefits of oat forage. Among oat consumption all over the world, feeding is the main, supplemented by food, and feeding consumption accounts for almost 60% (Marshall et al., 2013).

Historically, the Loess Plateau has been one of the key primary livestock production hubs, contributing significantly to the national livestock population with 12,311,000 cattle and 56,525,000 sheep, representing 11% and 19% of China’s total livestock, respectively (Zhang et al., 2017b). Thus, demand of forages in the Loess Plateau is on the rise due to market demand fueled by the animal industry. However, recent studies on forage crops in the region mainly focus corn and alfalfa (Jia et al., 2018; Zhang et al., 2017b; Kamran et al., 2022; Liu et al., 2021), and often neglects oats, a high-quality forage that is resistant to drought and infertility. Therefore, it is necessary to conduct further research on oats to fill this gap. Oat is grown by smallholder farmers focus on forage for livestock in this region. However, the agroecosystems of Loess Plateau in semi-arid regions are highly vulnerable due to limited available soil nutrient (Li et al., 2022; Wang et al., 2018), the growth of oat forages is usually negatively impacted by poor soil fertility. Maximizing the potential of oat cultivation, and improving oat nutrient efficiency crude protein (CP) yield in this region is a crucial strategy for addressing forage issues and fostering regional economic development.

Fertilization and planting density are two critical agronomic practices in cropping system for high productivity (Fu et al., 2021). Nitrogen is a limiting nutrient for plant growth and development (Gaudinier et al., 2018), while phosphorus is significant for plant development and reproduction (Zou et al., 2022). Both are essential macronutrients for improving agricultural productivity and maintaining ecosystem stability, and their combined application can lead to significant improvements in quality and yield of crop. Understanding the complex interactions between N and P use is critical for maximizing their nutrient efficiency in crops. Research indicates that their bilateral interaction promotes each other in the uptake and utilization of nutrition (Wang et al., 2020), and their combined application can enhance crop’s capacity to absorb nutrients (Zhang et al., 2021a). But the nutrient use efficiency varies with N and P supply levels, rapid nutrients loss occurs if the fertilizer rate is beyond the uptake capacity of crops. On the other hand, achieving high-yield and improved nutrients use efficiency under increasing planting density has been the priority goal of oat production. However, the higher planting densities may lead to increased competition among plants for nutrients, which may decrease the uptake of nitrogen and P, ultimately reduce protein and dry matter in the harvested crop. Hence, optimum fertilizer rate and planting density varies depending on field fertility condition as well as uptake capacity of crop, it is necessary to understand the mechanisms of nutrition use efficiency improvement of oats, which will in turn facilitate progress in management for field fertilizer.

The optimal fertilization rate coupled improving sowing density are the effective ways to improve crop productivity and maintain sustainable crop production (Yousaf et al., 2016). By focusing on the interplay between planting density and nutrient utilization can achieve better crop performance while minimizing negative environmental impacts (Hawkesford and Griffiths, 2019). Several studies have confirmed that improving planting density compatible with fertilizers application might benefit crop production for high yield with high fertilizer use efficiency. While, the improving planting density may compensate for the yield loss from fertilizer input reduction (Zhu et al., 2016; Huang et al., 2018). Notably, improving sole fertilizer application does not maximize the yield potential of crop population, while coupled appropriate planting density can significantly enhance the productivity of crop populations, resulting in increased yield of target crops (Wu et al., 2024). Moreover, enhancing nutrient efficiency through appropriate planting density coupled fertilizers not only contributes to greater nutrient accumulation but also supports sustainable agricultural practices by reducing the need for excessive fertilization. This is particularly important regarding the environmental concerns associated with fertilizers loss and soil degradation (Xu et al., 2012; Chen and Liao, 2017). In summary, the strategic management of sowing density, when coordinated with effective nutrients uptake and utilization practices, can lead to significant improvements in crop productivity and sustainability.

Moreover, yield and resource utilize efficiency of oat are affected by soil moisture, nutrients, light, and many other factors. The soil water and fertility conditions and agriculture systems are highly diverse in time and space, and the strategy of optimal sowing density and fertilization rate for another region may not be suitable for Loess Plateau (Kamran et al., 2023). Farmers in Loess Plateau of China are experiencing a lack of scientific recommendations of fertilizer application and reasonable sowing density for oats. They generally use intensive agronomic practices to increase the yield due to limited precipitation and soil degradation (Si et al., 2020; Tang et al., 2018). However, the negative impact on resource efficiency and the ecological environment is often ignored. Excess fertilizer amount and sowing density produce severe soil-ecological disruption and addition of cultivation cost, these intensive agronomic practices are untenable, both from economic and ecological benefit perspectives. To the best of our knowledge, there are very few reports from quality characteristics of oat in Loess Plateau, and even less about the response of nutrient efficiency to fertilizer and sowing density, thereby, little is known if forage yield, nutritive quality and resource efficiency of oat can simultaneously be improved with optimized fertilization and density in the Loess Plateau.

We hypothesized that the current fertilizers amount and plant density management for oat in the Loess Plateau is unreasonable and not conducive to cultivation of oat. Instead, optimizing fertilizer rate and sowing density would be valuable in improving yield, nutritional quality and nutrients use efficiency. Therefore, the present study was aimed to investigate the effects of different fertilizer rate and sowing density on forage yield, quality, nutrients indices and nutrients use efficiency for oat, and to determine a suitable management practice for maximize oat productivity in the Loess Plateau of northwest China. The results of this study would ensure the demands of taking full advantage of nutrient, while sustaining forage production and could provide new insights into the sustainable cultivation of oat on nutrient-limited land of Loess Plateau.

## 2 Materials and method

### 2.1 Experimental site details

Field experiments were conducted during 2022–2023 in Huan County, Gansu Province (36°16’ N, 107°31’ E; elevation, 1150 m), located in the Loess Plateau, Northwest China. The average annual precipitation in this region is 430 mm and 70% occurs during oat growing season (May to September). The annual precipitation is 414.5 and 364.6 mm in 2022 and 2023, the average temperature over oat growing season is 19.6 °C. The soil at the experimental site is classified as “loessial soil”. Basic soil properties were as follows: organic matter, available nitrogen, available phosphorus, available potassium and pH in the 0-20 cm soil layer was determined as 4.7 g kg^-1^, 54.1 mg kg^-1^, 11.3 mg kg^-1^, 166.4 mg kg^-1^ and 8.5, respectively.

### 2.2 Experimental design details

Field experiments were conducted to evaluate six fertilizer rates: N0P0, no fertilization; N2, 100 N kg/ha; P2, 90 P kg/ha; N1P2, 50 N kg/ha + 90 P kg/ha; N2P1, 100 N kg/ha + 45 P kg/ha; N2P2, 100 N kg/ha + 90 P kg/ha. Three sowing densities: L, 75 kg/ha; M, 150 kg/ha; H, 225 kg/ha. The combination of fertilizer rates and sowing densities were a total of eighteen treatments. In detail, the fertilizer rates and sowing densities were determined by considering both the soil base nutrition status and the farmers’ practices (namely, N2P2 treatment and H treatment; Zhang et al., 2025a). Fertilizer rates were divided into two gradients, increasing N fertilizer amount at the same P fertilizer level (N0P2, N1P2, N2P2); increasing P fertilizer amount at the same N fertilizer level (N2P0, N2P1, N2P2).

The experiment was conducted in a randomized complete block design with four replicates. Each plot covered an area of 24 m^2^ (4 × 6 m) and with a row spacing of 1 m, ridges were established between each adjacent plot to minimize runoff and fertilizer movement. Ridge cultivation of each plot was performed before sowing, fertilizer was applied in equal parts at the sowing (50%) and jointing (50%) stages. On 28th May 2022 and 18th May 2023, oat seeds (Baiyan No.7, provided by the Baicheng Academy of Agricultural Sciences) were manually sown at a soil depth of 4–5 cm with a 20 cm row spacing. Irrigation was not applied during the oat growing seasons, and weed and pest control were conducted manually. The harvesting of plants took place on 10th October 2022 and 5th October 2023, depending on the prevailing weather conditions.

### 2.3 Data collection and measurement

#### 2.3.1 Measurement of forage yield and quality

At heading and maturity stages of oat, three separate areas (each 1 m^2^) were randomly chosen from each plot, harvested aboveground oat plants and subjected to a drying process at 85°C until reaching a constant weight. The measured result of above dry matter accumulation was considered as forage yield.

Oven-dried oat samples were crushed into fine powder, passed through a 1-mm mesh screen, and prepared for the determination of forage quality and content of N and P. The concentration of crude protein (CP, %) was estimated by determining N content, and the N content was determined via Elementar Vario MAX CNS/CN (Elementar Trading Co., Ltd, Frankfurt, Germany).

The calculation equation (Equation 1) used for CP content were as follows (Zhang et al., 2018):


(1) CP content=N content×6.25

The concentrations of acid detergent fiber (ADF, %) and neutral detergent fiber (NDF, %) were determined following the methods of Vansoest et al. (1991).

#### 2.3.2 Calculation of forage feeding values

The relative feed value (RFV) of oat was calculated in maturity stage. The CP yield (kg ha^-1^) was calculated in heading and maturity stages. The equations (Equations 2, 3) used for calculation were as follows (Lithourgidis et al., 2006):


(2) RFV=(88.9−0.779×ADF)×(120/NDF)/1.29

(3) CP yield=aboveground dry matter yield×CP content

#### 2.3.3 Measurement of forage N and P content and uptake

N content (g kg^-1^) of oat in heading and maturity stages were determined via Elementar Vario MAX CNS/CN (producer information provided in section 2.3.1). Oat samples were digested with H_2_SO_4_-H_2_O_2_, and the P content (g kg^-1^) of oat at heading and maturity stages were determined with molybdate blue colorimetry as described by (Cao et al., 2021).

Total N and P uptake (kg ha^-1^) of heading and maturity stages were calculated according to aboveground dry weight (t ha^-1^) and N/P content (Equations 4, 5):


(4) N uptake=N content×aboveground dry matter yield

(5) P uptake=P content×aboveground dry matter yield

#### 2.3.4 Calculation of forage N and P efficiency

The fertilizer agronomic efficiency (AE), N uptake efficiency (NupE), P uptake efficiency (PupE), N recovery efficiency (NRE) and P recovery efficiency (PRE) of oat was calculated in maturity stage and according to forage yield and N/P uptake of maturity stage.

The calculation formula (Equations 6–10) as follows (Yang et al., 2024):


(6) $$AE=\frac{forage\;yield-forage\;yield\;of\;no\;fertilizer}{Total\;fertilizer\;input}$$

(7) NupE=N uptake/ input of N

(8) PupE=P uptake/ input of P

(9) NRE=(N uptake−N uptake of no fertilizer)/ input of N

(10) PRE=(P uptake−P uptake of no fertilizer)/ input of P

#### 2.3.5 Comprehensive evaluation of N and P

Membership function analysis was implemented for comprehensive evaluation of N and P. The N evaluation indicator include N content of heading stage, N content of maturity stages, N uptake of heading stage, N uptake of maturity stages, NupE and NRE; the P evaluation indicator include P content of heading stage, P content of maturity stages, P uptake of heading stage, P uptake of maturity stages, PupE and PRE. The comprehensive analysis score of each indicator of nitrogen was defined as “NCS (nitrogen comprehensive score)”, and the comprehensive analysis score of each indicator of phosphorus was defined as “PCS (phosphorus comprehensive score)”. The optimal fertilization and density treatment was evaluated by result of analysis.

The calculation method Equations 11, 12 of the comprehensive evaluation indicator was as follows (Li et al., 2023):


(11) $$NCS=\sum^{}MN_{1}W_{1}+MN_{2}W_{2}+\ldots +MN_{n}W_{n}$$

(12) $$PCS=\sum^{}MP_{1}W_{1}+MP_{2}W_{2}+\ldots +MP_{n}W_{n}$$

where *MN* is the N indicator’s membership value, *MP* is the P indicator’s membership value, and *W* is the weighting factor. The weightings of each indicator were the same at one in six in this study.

The membership values were counted as follows (Equation 13):


(13) $$M\left(X_{i}\right)=\left(X_{i}-X_{min}\right)/\left(X_{max}-X_{min}\right)$$

where *M(Xi)* is the membership value, and *M(Xi)* ∈[0,1]; *Xi* is each index’s measured value; *Xmin* and *Xmax* are each index’s minimum and maximum values.

### 2.4 Statistical analysis

Statistical data analysis was performed using analysis of variance (ANOVA) in SPSS statistics software 27.0 (IBM Corporation, USA). Multiple comparisons between different treatments were conducted using the Tukey’s significant test that significance level of *P*< 0.05. The relationship and interactions of measured variables assessed by Pearson’s correlation and principal component analysis (PCA). Figures were used via Origin 2019 (Origin Lab Corporation, USA).

## 3 Result

### 3.1 Nutritive quality of oat

**Figure 1:**
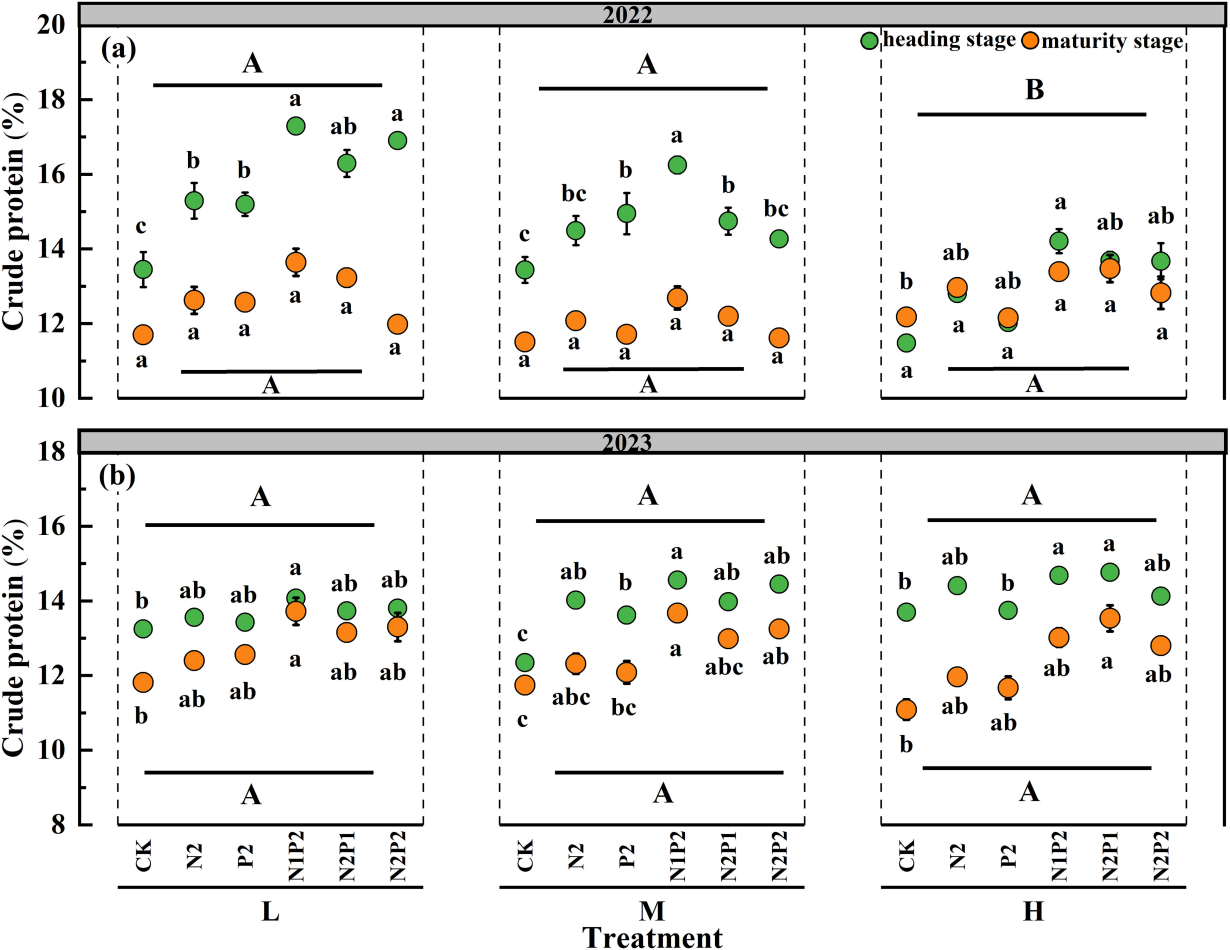
Variations in oat crude protein under different sowing densities (SD) and fertilizer rates (FR) at heading and maturity stages in 2022 **(a)** and 2023 **(b)**. L, low sowing density; M, moderate sowing density; H, high sowing density. Vertical bars represent SE values (*P*< 0.05). Different lower-case letters above or under the circles denote significant differences for different fertilizer rates under the same sowing density (*P*< 0.05). Different upper-case letters in top or bottom of figure indicate significant differences between sowing densities (*P*< 0.05).

The statistical results revealed that the sowing density (SD) had no significant impact on oat crude protein (CP) content except heading stage of 2022. However, the effect of fertilizer rate (FR) on CP was significant (*P*< 0.05) in all stages of two years. The interaction effect of the SD and FR had no significant impact on the CP content (**Supplementary Table S1**). The CP content was increased with fertilizer application in the two growth seasons, and the highest average CP content of three sowing densities (15.47 and 14.12%) were obtained under the N1P2 treatment, markedly increased by 23.1 and 17.2% in 2022 and 2023, respectively, compare with that in the no fertilization treatment (**Figures 1a, b**).

**Figure 2:**
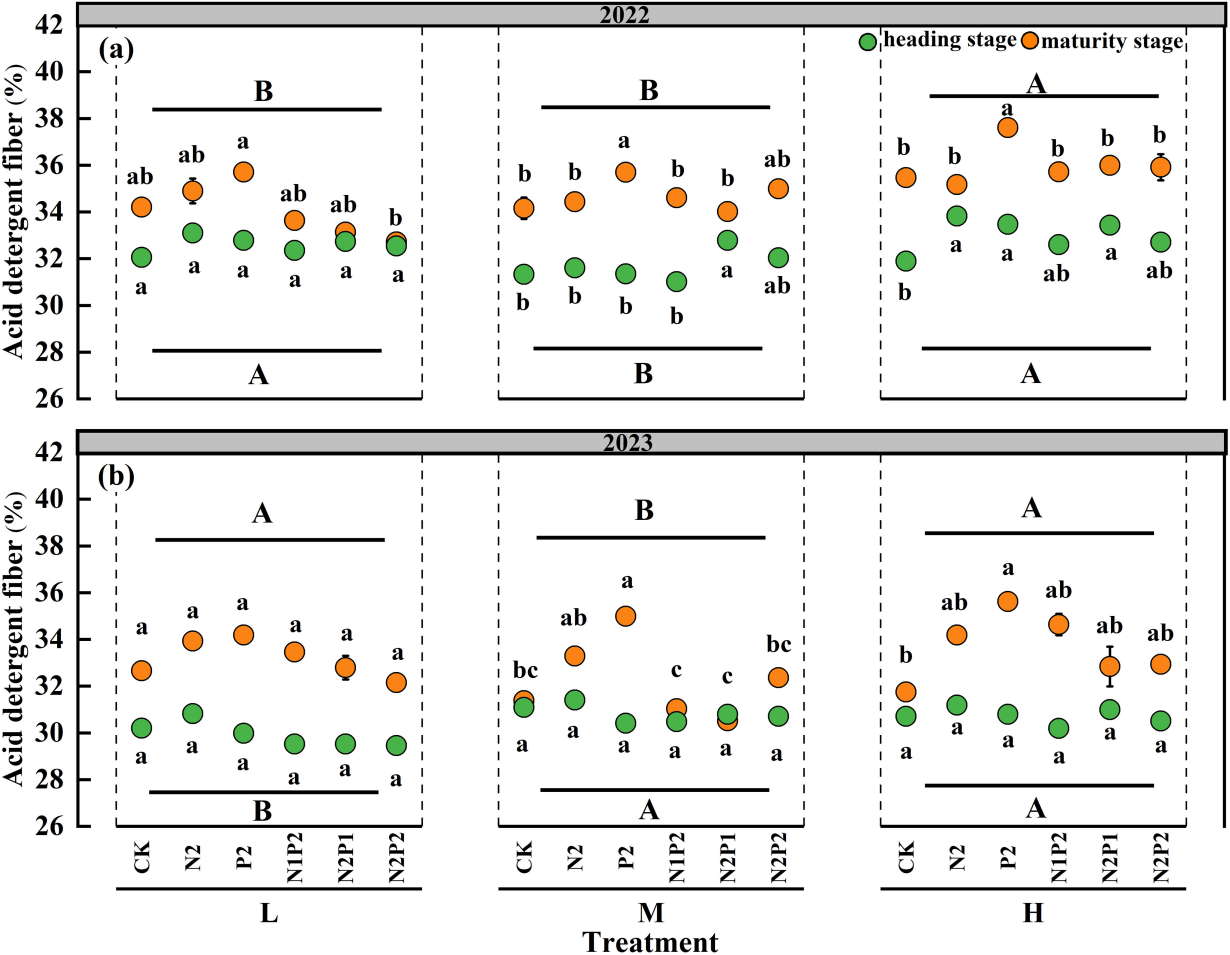
Variations in acid detergent fiber under different sowing densities (SD) and fertilizer rates (FR) at heading and maturity stages in 2022 **(a)** and 2023 **(b)**. L, low sowing density; M, moderate sowing density; H, high sowing density. Vertical bars represent SE values (*P*< 0.05). Different lower-case letters above or under the circles denote significant differences for different fertilizer rates under the same sowing density (*P*< 0.05). Different upper-case letters in top or bottom of figure indicate significant differences between sowing densities (*P*< 0.05).

The SD had significant (*P*< 0.05) effect on acid detergent fiber (ADF) of oat, the FR had significant (*P*< 0.05) impact on oat ADF except heading stage of 2023, and the interaction effect of the SD and FR had no significant imapct on the ADF during two years (**Supplementary Table S1**). A lower ADF was observed under the M treatment than that under the L and H treatments in heading stage of 2022 and maturity stage of 2023 (**Figures 2a, b**). The FR more markedly affected the ADF at the maturity stage compare with that at the heading stage (**Figures 2a, b**).

**Figure 3:**
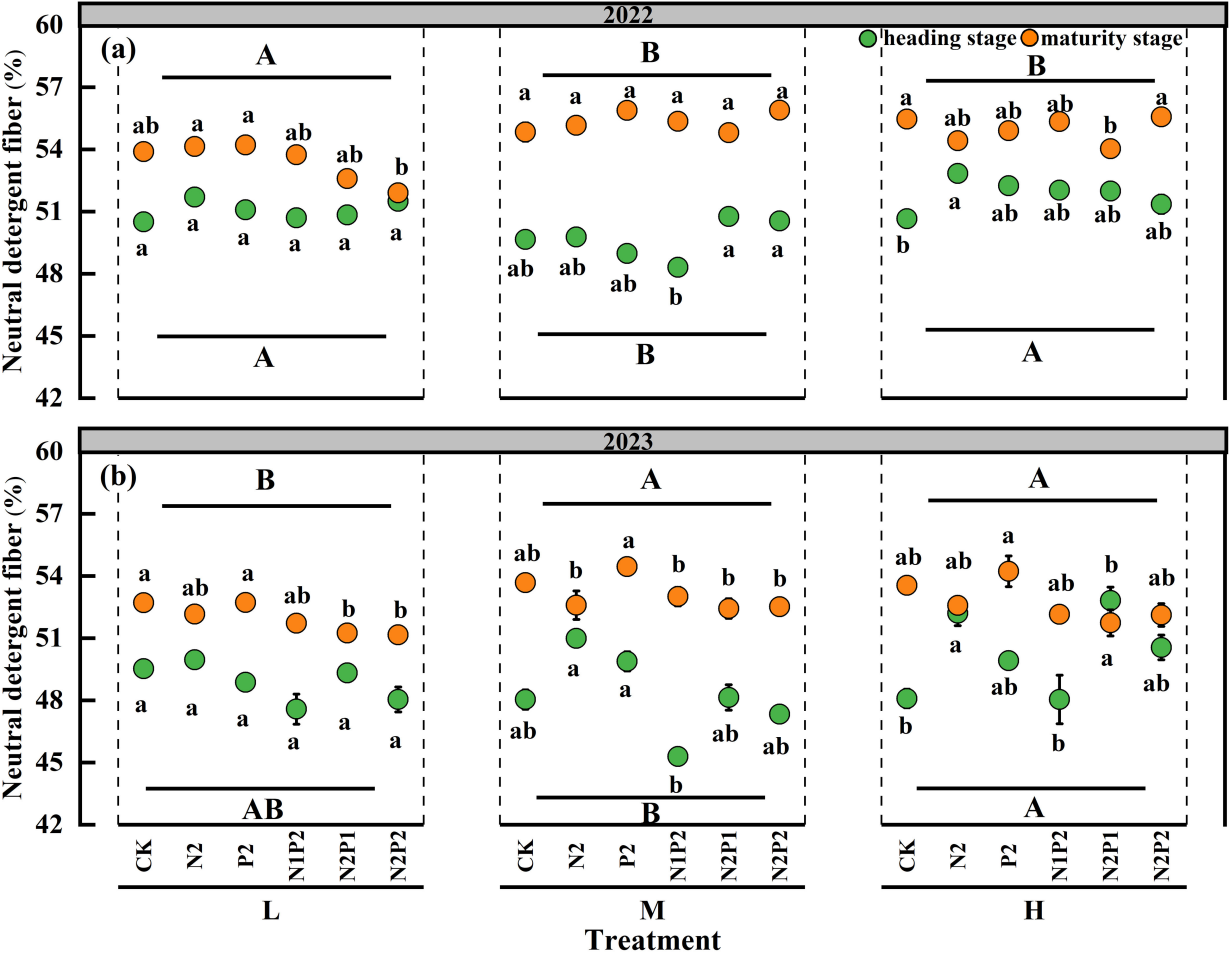
Variations in neutral detergent fiber under different sowing densities (SD) and fertilizer rates (FR) at heading and maturity stages in 2022 **(a)** and 2023 **(b)**. L, low sowing density; M, moderate sowing density; H, high sowing density. Vertical bars represent SE values (*P*< 0.05). Different lower-case letters above or under the circles denote significant differences for different fertilizer rates under the same sowing density (*P*< 0.05). Different upper-case letters in top or bottom of figure indicate significant differences between sowing densities (*P*< 0.05).

The effects of the SD and FR on the neutral detergent fiber (NDF) of oats were significant (*P*< 0.05) in both years, but the interaction effect of the SD and FR had no significant impact on the NDF (**Supplementary Table S1**). The M treatment significantly decreased the NDF except maturity stage of 2023. At the M treatment, compared with that in the no fertilization treatment, the NDF under the N1P2 was significantly decreased by 1.7 and 3.5% in 2022 and 2023, respectively (**Figures 3a, b**).

The CP, ADF and NDF significantly differed between the heading and maturity stages. Compared with those in the heading stage, the average CP in the two years were decreased 13.0% in the maturity stage, while the average ADF and NDF in the two years were increased 8.0% and 7.2% in the maturity stage (**Figures 1**-**3**).

**Figure 4:**
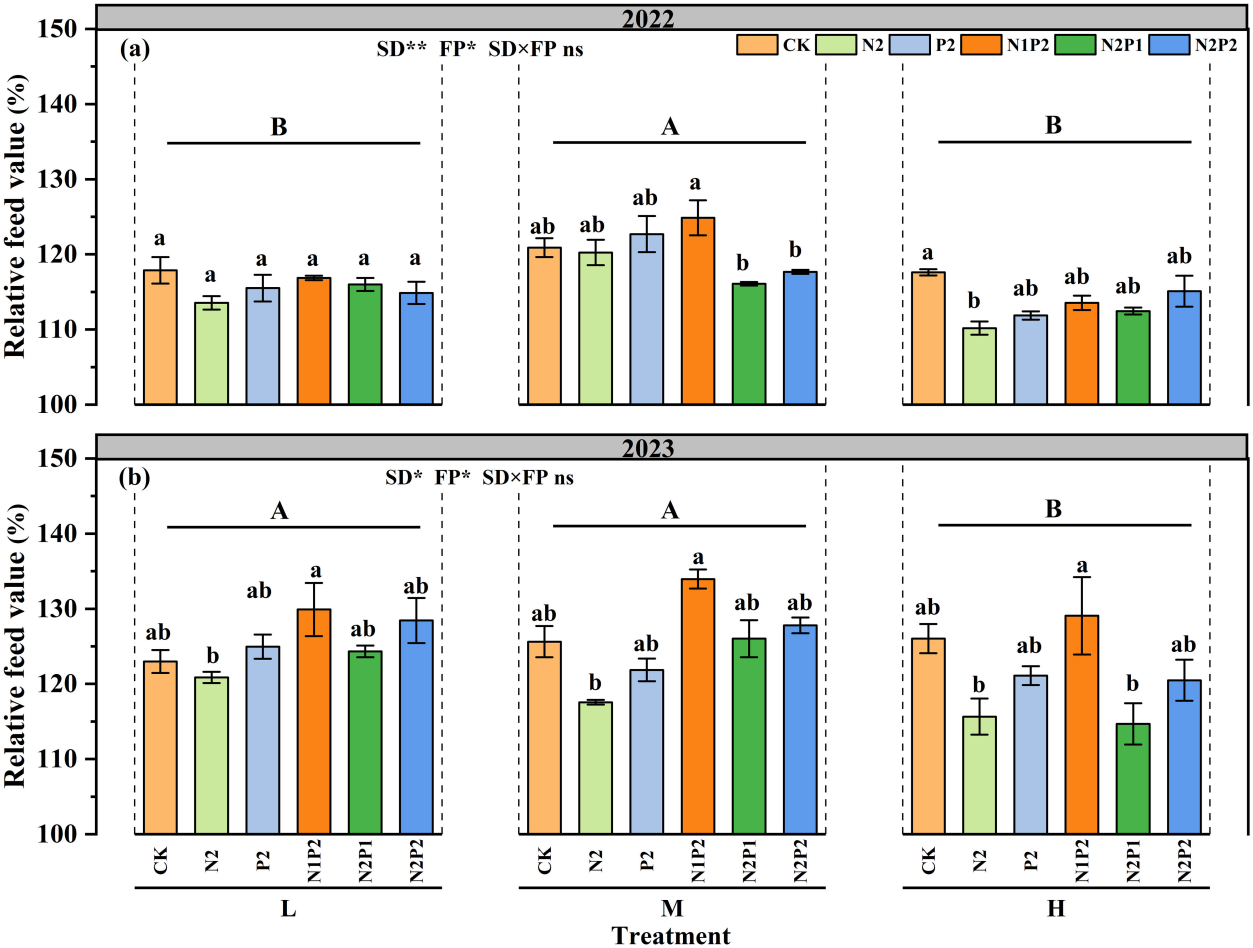
Variations in relative feed value under different sowing density (SD) and fertilizer rates (FR) during 2022 **(a)** and 2023 **(b)**. L, low sowing density; M, moderate sowing density; H, high sowing density. Vertical bars represent SE values (*P*< 0.05). Different lower-case letters above the bars denote significant differences for different fertilizer rates under the same sowing density (*P*< 0.05). Different upper-case letters indicate significant differences between sowing density (*P*< 0.05).

Different SD and FR treatments significantly (*P*< 0.05) affected the relative feed value (RFV) but their interaction was not. At the M treatment, compare with that in the no fertilization treatment, the RFV values peaking at N1P2 and increasing by 3.2 and 6.6% in 2022 and 2023, respectively (**Figures 4a, b**).

### 3.2 Forage yield, crude protein yield and agronomic efficiency

The SD, FR and their interaction effect significantly (*P*< 0.05) affected the forage yield and CP yield of oats (**Table 1**). Compared with that in the M treatment, the forage yield under L and H treatments were decreased by 18% and 23.9% in 2022 and 34.3% and 46.1% in 2023, respectively. CP yield also reached a maximum at M treatment, and reduced or increase sowing density treatments showed detrimental effects on CP yield. Compare with no fertilizer treatment, the average forage yield of two stages was markedly increased 54.5 and 91.8% under the N1P2 treatment in 2022 and 2023, respectively. The average CP yield showed the same trend with the change of forage yield, peaked at the N1P2 treatment and increasing by 81.2 and 118.8% in 2022 and 2023, respectively, compare with that under the no fertilization treatment. The heading stage had a higher CP content than maturity stage, but maturity stage obtained more forage yield and CP yield, these trends remained steady throughout the experimental duration (**Table 1**).

**Table 1:** Forage yield, crude protein (CP) yield and agronomic efficiency (AE) of oat at 2022 and 2023.

Years	Sowing densities	Fertilizer rates	Forage yield (t ha^-1^)		CP yield (kg ha^-1^)		AE (kg kg^-1^)
			Heading stage	Maturity stage	Heading stage	Maturity stage	
2022	L	CK	2.97b	4.58b	405.11b	522.96b	
		N2	3.15ab	5.18ab	485.55b	652.44ab	5.96b
		P2	3.15ab	4.91ab	477.12b	623.45ab	3.59b
		N1P2	4.07a	6.43a	706.82a	890.99a	13.2a
		N2P1	3.38ab	5.81ab	550.95ab	765.72ab	8.45ab
		N2P2	3.07ab	5.42ab	519.31ab	621.79ab	4.40b
		**Mean**	**3.30B**	**5.39B**	**524.14B**	**679.56B**	**7.12B**
	M	CK	3.77b	5.60c	503.39b	633.44b	
		N2	4.37ab	6.69b	641.83b	774.87b	10.9b
		P2	3.80b	6.49c	570.80b	642.11b	9.92b
		N1P2	6.35a	8.13a	1021.07a	1037.56a	18.09a
		N2P1	4.96ab	6.85b	764.01ab	871.04ab	8.63b
		N2P2	4.27ab	7.34ab	604.89b	849.87ab	9.2b
		**Mean**	**4.59A**	**6.68A**	**684.33A**	**801.48A**	**10.41A**
	H	CK	3.25b	4.53b	364.72b	539.34b	
		N2	4.52a	6.00ab	601.91ab	767.86ab	14.62a
		P2	4.10a	5.04ab	484.13b	611.23ab	5.58b
		N1P2	4.74a	6.79a	656.92a	906.97a	16.14a
		N2P1	4.54a	5.93ab	623.67a	827.51ab	9.65ab
		N2P2	4.21a	5.66ab	584.60ab	710.49ab	5.93b
		**Mean**	**4.23A**	**5.66B**	**552.66B**	**727.23AB**	**10.38A**
	ANOVA	SD	**	**	*	*	*
		FR	**	**	**	**	**
		SD*FR	*	*	*	*	ns
2023	L	CK	2.29b	3.35c	306.83b	396.07c	
		N2	3.07ab	5.31ab	414.54bc	649.08bc	19.61a
		P2	2.56b	4.59bc	344.16b	572.07bc	13.75b
		N1P2	3.95a	6.06a	556.35a	876.18a	19.36a
		N2P1	3.32ab	5.32ab	453.90ab	696.06ab	13.57b
		N2P2	3.29ab	5.36ab	453.76ab	700.72ab	10.55b
		**Mean**	**3.08B**	**5.00B**	**421.59B**	**648.36B**	**15.4AB**
	M	CK	2.58c	5.34bc	319.65c	634.63b	
		N2	3.38bc	5.85bc	477.92bc	690.29b	5.11c
		P2	2.78c	5.87c	380.95c	540.76b	5.64c
		N1P2	4.91a	10.28a	710.90a	1376.17a	35.31a
		N2P1	4.67ab	9.35ab	651.81ab	1215.36a	27.68ab
		N2P2	4.17abc	8.43abc	604.07ab	1108.48a	16.24b
		**Mean**	**3.75A**	**7.29A**	**524.22A**	**927.61A**	**17.2A**
	H	CK	3.18b	4.20ab	439.47b	493.24b	
		N2	3.31b	4.95ab	476.14b	586.23b	7.53b
		P2	3.41b	4.69b	470.56b	441.79b	5.44b
		N1P2	4.61a	7.75a	674.75a	1044.79a	25.40a
		N2P1	4.37a	7.46ab	644.13a	1026.84a	22.47a
		N2P2	3.24b	4.53ab	448.76b	584.79b	1.74c
		**Mean**	**3.69A**	**5.43B**	**525.64A**	**696.28B**	**11.76B**
	ANOVA	SD	*	**	*	**	*
		FR	**	**	**	**	*
		SD*FR	*	*	*	*	ns

Data represent the mean. Different lower-case letters denote significant differences within same sowing density (SD) for different fertilizer rates (FR; *P*< 0.05). Different upper-case letters denote significant differences for different sowing densities (*P*< 0.05).

L means low sowing density; M means moderate sowing density; H means high sowing density.

ANOVA indicates analysis of variance; ** indicates *P*< 0.01; *indicates *P*< 0.05; ns indicates no significance.

Agronomic efficiency (AE) of oats was significantly (*P*< 0.05) affected by SD and FR but not their interaction effect (**Table 1**). The average AE under M treatment was greater than with other density treatments of two years. At the M density, compare with that in the other fertilization treatments, the N1P2 treatment significantly increased the AE by 66.0–109.6% and 27.6–390.8% in 2022 and 2023, respectively (**Table 1**).

### 3.3 Content, uptake, efficiency and comprehensive score of nitrogen

**Figure 5:**
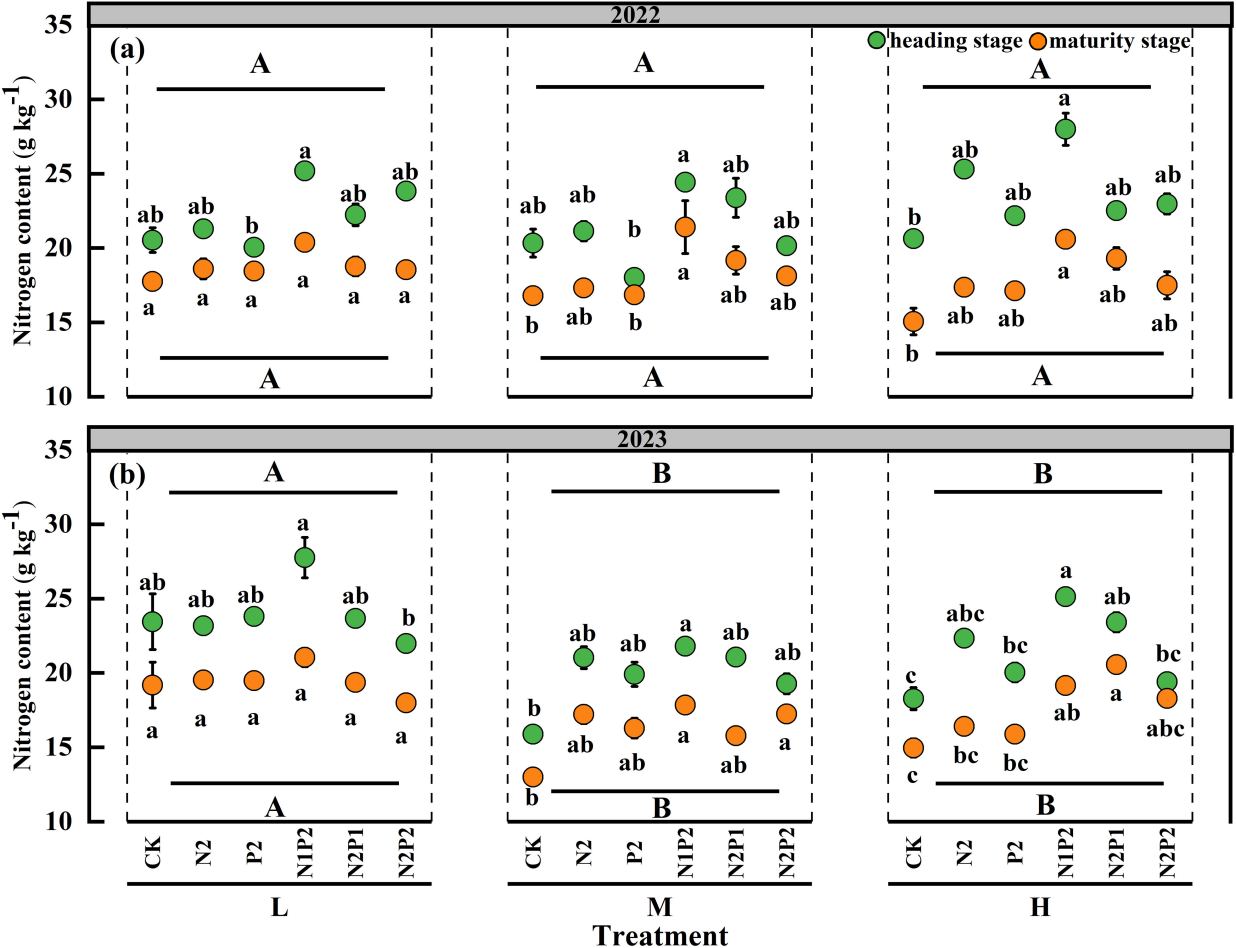
Variations in oat N content under different sowing density (SD) and fertilizer rates (FR) at heading and maturity stages in 2022 **(a)** and 2023 **(b)**. L, low sowing density; M, moderate sowing density; H, high sowing density. Vertical bars represent SE values (*P*< 0.05). Different lower-case letters above or under the circles denote significant differences for different fertilizer rates under the same sowing density (*P*< 0.05). Different upper-case letters in top or bottom of figure indicate significant differences between sowing densities (*P*< 0.05).

In both oat growing seasons, there were significant (*P*< 0.05) differences in N content among the FR treatments, the SD had significant (*P*< 0.05) effect on the N content only in 2023, the interaction effect of the SD and FR had no significant impact on the N content (**Supplementary Table S2**). The N content of oats markedly increased with the fertilization, and the maximum values were achieved under the N1P2 treatment in all seasons of two years. Under the N1P2 treatments, the average N content of two stages was significantly increased by 23.4% in 2022 and 37.3% in 2023 than that in the no fertilization treatment, respectively. Compared with FR treatment, the effect of SD treatment on N content was less. The SD treatments had no significant effects on the N content in 2022. Compared with that in the M and H treatments, the N content increased under the L treatment in 2023. During oat growing seasons, the N content decreased in the maturity stage compare with heading stage (**Figures 5a, b**).

Statistically, FR, SD and their interaction effect had a significant (*P*< 0.05) impact on N uptake of oats. The M treatment significantly increased the N uptake in maturity stage of two years, as showed in the **Table 2**. The N uptake markedly increased with the fertilization and peaked at N1P2 treatment in all stages of both years. At the M treatment, the average N uptake of two stages in the N1P2 treatment increased by 94.0 and 162.9% in 2022 and 2023, respectively, compared with that in the no fertilizer treatment (**Table 2**). Although the heading stage had greater N content of oat, the maturity stage obtained greater N uptake because of more forage yield (**Figure 5**; **Table 2**).

**Table 2:** Total N uptake (kg ha^-1^), N uptake efficiency (NupE, kg ha^-1^) and N recovery efficiency (NRE, kg ha^-1^) of oat.

SD	FR	2022				2023			
		N uptake		NupE	NRE	N uptake		NupE	NRE
		Heading stage	Maturity stage			Heading stage	Maturity stage		
L	CK	58.65b	79.33b			57.18b	65.13c		
	N2	66.95b	96.58b	0.97b	0.17b	71.05b	101.75ab	1.02b	0.37b
	P2	62.81b	91.40b			61.17b	89.27bc		
	N1P2	103.00a	127.00a	2.54a	0.95a	111.84a	128.86a	2.58a	1.27a
	N2P1	77.46b	107.23ab	1.07b	0.28b	77.50b	106.81ab	1.07b	0.42b
	N2P2	73.27b	95.13b	0.95b	0.16b	71.87b	95.32bc	0.95b	0.30b
	**Mean**	**73.69B**	**99.44B**	**0.92B**	**0.26B**	**75.10B**	**97.86B**	**0.94B**	**0.39B**
M	CK	73.72c	92.52c			40.37c	68.07d		
	N2	91.67bc	111.70bc	1.12b	0.19b	69.10bc	96.91bc	0.97b	0.29c
	P2	68.07c	92.49c			63.47bc	73.43cd		
	N1P2	153.38a	174.99a	3.50a	1.65a	99.30a	185.79a	3.72a	2.35a
	N2P1	117.11b	136.84ab	1.37b	0.44b	100.35a	143.52ab	1.44b	0.75b
	N2P2	86.50bc	134.99ab	1.35b	0.42b	76.83ab	143.49ab	1.43b	0.75b
	**Mean**	**98.41A**	**123.92A**	**1.22A**	**0.45A**	**74.90B**	**118.53A**	**1.26A**	**0.69A**
H	CK	65.84c	65.14c			59.01c	61.50b		
	N2	115.09bc	101.90bc	1.02b	0.37b	78.00bc	80.71b	0.81b	0.19b
	P2	89.25bc	87.07bc			74.08bc	60.96b		
	N1P2	133.38a	136.83a	2.74a	1.43a	117.69a	153.49a	3.07a	1.84a
	N2P1	101.47b	113.12ab	1.13b	0.48b	100.65ab	153.86a	1.54b	0.92b
	N2P2	97.34bc	97.23bc	0.97b	0.32b	73.38bc	85.56b	0.86b	0.24b
	**Mean**	**100.39A**	**100.21B**	**0.98B**	**0.43A**	**83.80A**	**99.35B**	**1.05B**	**0.53AB**
ANOVA	SD	*	*	*	*	*	*	*	*
	FR	**	**	**	**	*	**	**	**
	SD*FR	*	*	ns	ns	*	*	ns	ns

Data represent the mean. Different lower-case letters denote significant differences within same sowing density (SD) for different fertilizer rates (FR; *P*< 0.05). Different upper-case letters denote significant differences for different sowing densities (*P*< 0.05).

L means low sowing density; M means moderate sowing density; H means high sowing density.

ANOVA indicates analysis of variance; ** indicates *P*< 0.01; * indicates *P*< 0.05; ns indicates no significance.

The FR and SD treatments had significant effects on nitrogen uptake efficiency (NupE) and nitrogen recovery efficiency (NRE). The highest NupE and NRE were obtained in the M-N1P2 treatment. At the M treatments, N1P2 increased NupE and NRE by 155.5–212.5% and 275–768.4% in 2022 and 158.3–283.5% and 213.3–710.3% in 2023, respectively, compared with those in the other treatments (**Table 2**).

**Figure 6:**
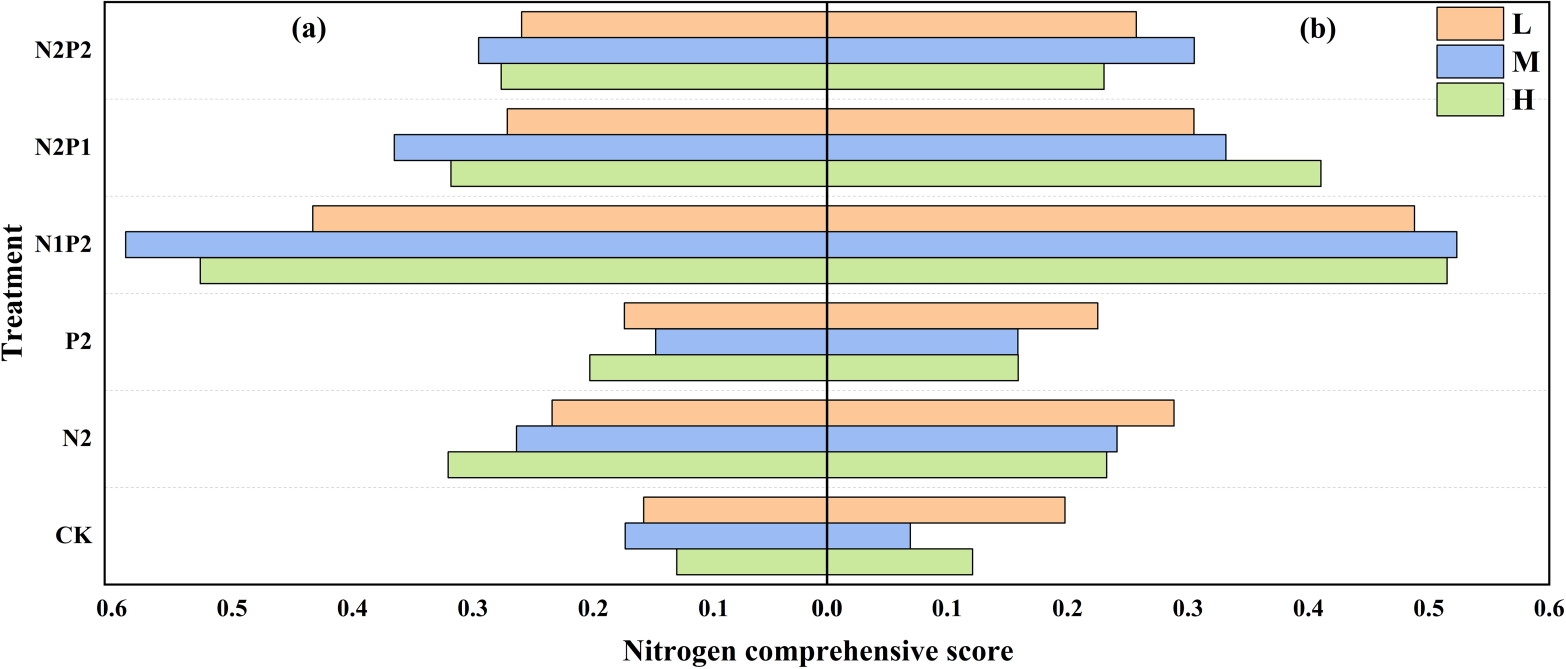
Variations in oat N comprehensive score under different sowing density (SD) and fertilizer rates (FR) in 2022 **(a)** and 2023 **(b)**. L, low sowing density; M, moderate sowing density; H, high sowing density.

In the evaluation system of N, a single indicator might produce a random error for evaluation result, so it was important to combine multiple indicators for their comprehensive evaluation to obtain more objective and accurate evaluation results. The indicators of N comprehensive evaluation include N content and N uptake of heading and maturity stages, NupE and NRE. The result of N comprehensive evaluation showed that N1P2 has the highest N comprehensive score (NCS) among all treatments (**Figure 6**).

### 3.4 Content, uptake, efficiency and comprehensive score of phosphorus

**Figure 7:**
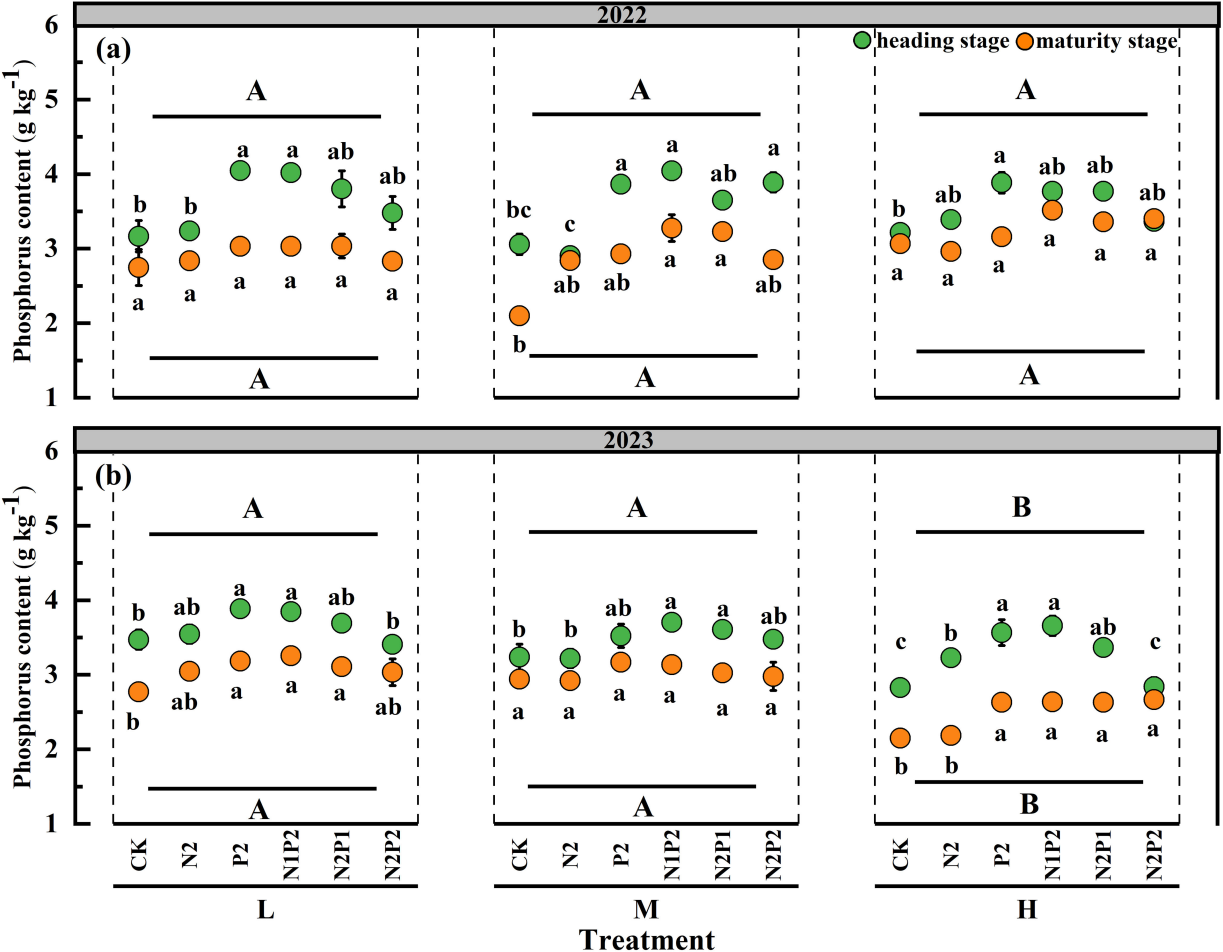
Variations in oat P content under different sowing density (SD) and fertilizer rates (FR) at heading and maturity stages in 2022 **(a)** and 2023 **(b)**. L, low sowing density; M, moderate sowing density; H, high sowing density. Vertical bars represent SE values (*P*< 0.05). Different lower-case letters above or under the circles denote significant differences for different fertilizer rates under the same sowing density (*P*< 0.05). Different upper-case letters in top or bottom of figure indicate significant differences between sowing densities (*P*< 0.05).

The results revealed that the FR had significant (*P*< 0.05) effect on the P content in both years, the SD had significant (*P*< 0.05) effect on the P content only in 2023, and their interaction had no significant effect on the P content (**Supplementary Table S2**). The P content of oats exhibited a similar trend in both years and markedly increased with the fertilization. P2 and N1P2 treatment were significantly increased the P content compared with that in the other fertilization. Under the M treatment, the N1P2 slightly increased P content by 41.9% and 10.7% in 2022 and 2023, respectively, compared with that in the no fertilization treatment. The differences between SD treatments were not statistically significant for P content in 2022, however, the H treatment decreased the P content in 2023 (**Figures 7a, b**).

The **Table 3** showed that the FR, SD and their interaction had significant affect (*P*< 0.05) on P uptake and efficiency. The P uptake increased with the fertilization and peaked at the M-N1P2 treatment in both two years. At the M treatment, the average P uptake of two stages in the N1P2 treatment increased by 128.1% and 108.6% compared with that in the no fertilizer treatment in 2022 and 2023, respectively (**Table 3**). Although the heading stage had higher P content of oat, the increase of forage yield led to more P uptake in maturity stage (**Figure 7**; **Table 3**).

**Table 3:** Total P uptake (kg ha^-1^), P uptake efficiency (PupE, kg ha^-1^) and P recovery efficiency (PRE, kg ha^-1^) of oat.

SD	FR	2022				2023			
		P uptake		PupE	PRE	P uptake		PupE	PRE
		Heading stage	Maturity stage			Heading stage	Maturity stage		
L	CK	8.97c	12.52c			7.49b	9.30b		
	N2	10.25bc	14.73bc			10.92ab	15.84ab		
	P2	12.73b	15.00b	0.17b	0.03b	10.00ab	14.52ab	0.16b	0.06c
	N1P2	16.42a	19.32a	0.21b	0.08ab	14.94a	20.32a	0.23b	0.12b
	N2P1	13.08ab	17.40ab	0.39a	0.11a	12.24ab	17.15ab	0.38a	0.17a
	N2P2	10.61bc	14.38bc	0.16b	0.02b	11.17ab	16.23ab	0.18b	0.08c
	**Mean**	**12.01B**	**15.56B**	**0.15B**	**0.04B**	**11.13B**	**15.56B**	**0.16B**	**0.07A**
M	CK	11.23c	11.64c			8.80c	15.45c		
	N2	12.82bc	18.24bc			10.85bc	16.35c		
	P2	14.71bc	16.08bc	0.18c	0.05c	11.07bc	13.45c	0.15c	-0.02c
	N1P2	25.30a	26.86a	0.30b	0.17ab	17.72a	32.86a	0.37b	0.19b
	N2P1	18.83ab	23.06ab	0.51a	0.25a	17.26a	28.33ab	0.63a	0.29a
	N2P2	16.34bc	20.97b	0.23bc	0.10bc	14.44ab	24.63bc	0.27c	0.10c
	**Mean**	**16.54A**	**19.47A**	**0.20A**	**0.10A**	**13.36A**	**21.85A**	**0.24A**	**0.09A**
H	CK	10.26b	13.34c			9.51c	8.96c		
	N2	15.40ab	17.89abc			11.30bc	11.30b		
	P2	15.69ab	15.67bc	0.17b	0.03c	12.91bc	9.77c	0.11b	0.01c
	N1P2	17.64a	24.09a	0.27b	0.12b	17.05a	20.41a	0.23b	0.13b
	N2P1	17.15a	20.77ab	0.46a	0.17a	14.72ab	19.61a	0.44a	0.24a
	N2P2	14.23ab	18.61abc	0.21b	0.06c	10.33c	12.50b	0.14b	0.04c
	**Mean**	**15.06A**	**18.40AB**	**0.19A**	**0.06AB**	**12.64AB**	**13.76B**	**0.15B**	**0.07A**
ANOVA	SD	**	*	*	*	*	**	**	ns
	FR	**	**	**	**	*	**	**	**
	SD*FR	*	*	ns	ns	*	*	ns	ns

Data represent the mean. Different lower-case letters denote significant differences within same sowing density (SD) for different fertilizer rates (FR; *P*< 0.05). Different upper-case letters denote significant differences for different sowing densities (*P*< 0.05).

L means low sowing density; M means moderate sowing density; H means high sowing density.

ANOVA indicates analysis of variance; ** indicates *P*< 0.01; * indicates *P*< 0.05; ns indicates no significance.

**Figure 8:**
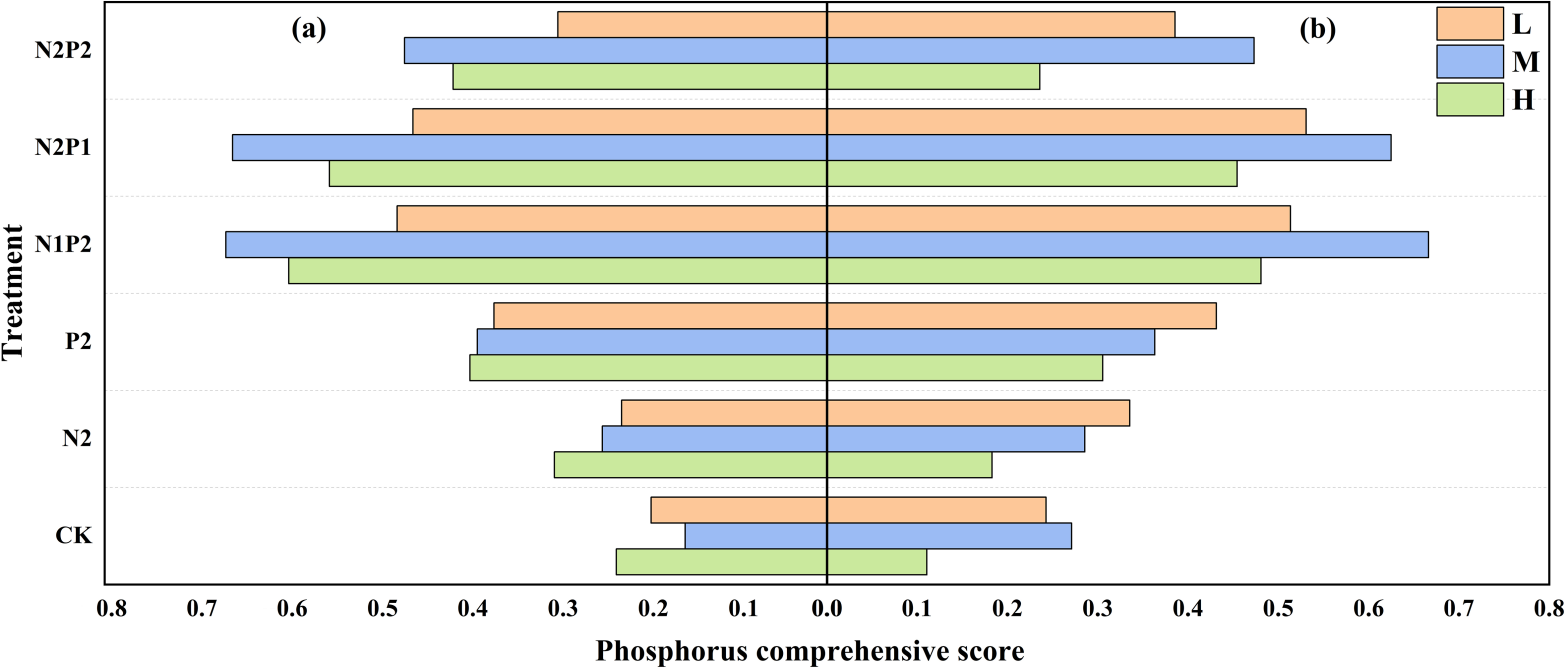
Variations in oat P comprehensive score under different sowing density (SD) and fertilizer rates (FR) in 2022 **(a)** and 2023 **(b)**. L, low sowing density; M, moderate sowing density; H, high sowing density.

The FR and SD treatments had significant effects on phosphorus uptake efficiency (PupE) and phosphorus recovery efficiency (PRE), the N2P1 treatment has the highest PupE and PRE, however, membership function analysis revealed that the highest P comprehensive score (PCS) under the N1P2 treatment (**Figure 8**).

Overall, the optimum combination of SD and FR (M-N1P2) for maximizing oat N/P content, uptake and comprehensive efficiency was the same in both years. Integrated nutrient management comprising N/P fertilizers in combined was better than solely applying fertilizer (**Figures 5**-**8**; **Tables 2**, **3**).

### 3.5 Comprehensive evaluation analysis

**Figure 9:**
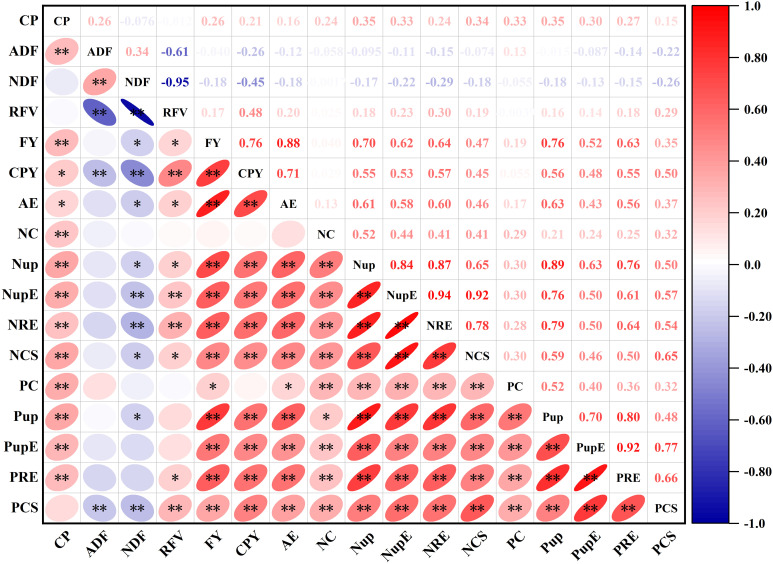
Correlation analysis for the correlations between quality, yield, nutrient efficiency and comprehensive score of oat forage. The variables included CP, crude protein; ADF, acid detergent fiber; NDF, neutral detergent fiber; RFV, relative feed value; FY, forage yield; CPY, crude protein yield; AE, agronomic efficiency; NC, nitrogen content; Nup, nitrogen uptake; NupE, nitrogen uptake efficiency; NRE, nitrogen recovery efficiency; NCS, nitrogen comprehensive score; PC, phosphorus content; Pup, phosphorus uptake; PupE, phosphorus uptake efficiency; PRE, phosphorus recovery efficiency; PCS, phosphorus comprehensive score. The color gradient denotes correlation coefficient. * and **represent the significant differences at P< 0.05 and P< 0.01, respectively.

The correlation analysis revealed significant positive correlations of forage yield, CP yield, nitrogen content and RFV with nutrient uptake and use efficiency (AE, NupE, NRE, NCS, PupE, PRE and PCS) of oat, but negative relations with ADF and NDF content. The CP content and phosphorus content followed a strong positive relationship with other variables except NDF. In addition, the correlation analysis indicated a significant positive relationship between ADF and NDF (**Figure 9**).

**Figure 10:**
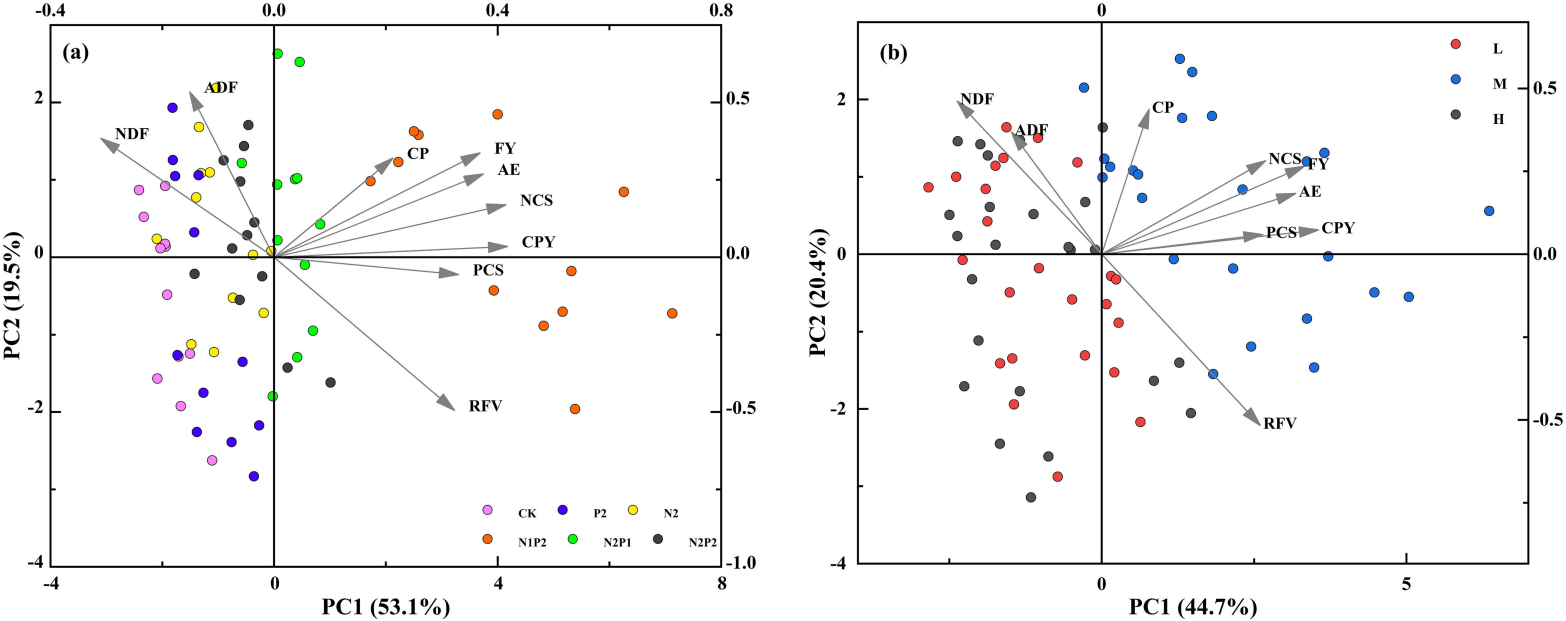
The principal component analysis (PCA) shows the relationship between variables and represents the separation of fertilizer rate treatments **(a)** and sowing density treatments **(b)** among the first two principal components. Abbreviations for indicator names are similar to **Figure 9**.

The principal component analysis (PCA) was prepared to present the concerted information on the forage yield and quality traits, nutrient content, uptake and use efficiency in relation to FR and SD treatments. For fertilizer rate (**Figure 10a**), the first two principal components explained 72.6% of the total variance (being 53.1% in PC1 and 19.5% in PC2). The result revealed the clear segregation of the variables and fertilizer treatments into different quadrants. The upper left quadrant of the negative side of PC1 included the CK, solely applying fertilizer (N2 and P2) and N2P2 treatment that delivered high ADF and NDF contents (**Figure 10a**). The upper right quadrant and lower right quadrant included N1P2 and N2P1 treatment representing higher forage yield, nutrient content, feed value, nutrient uptake and nutrient efficiency along with premium forage quality of oats. Finally, CK, N2, P2 and N2P2 treatments in left quadrant had the lower yield, quality, and nutrient content and efficiency, but higher ADF and NDF (**Figure 10a**). For sowing density (**Figure 10b**), the first two principal components explained 65.1% of the total variance (being 44.7% in PC1 and 20.4% in PC2). The L and H treatments clustered on the upper left and lower left quadrants that depicted the lower yield, quality, nutritional indictors and higher fiber content. The M treatment in right quadrants had the higher yield, quality, feed value and nutritional indictors (**Figure 10b**).

## 4 Discussion

### 4.1 Oat nutrient quality, N/P content and feed value response to fertilization and density

For forage producers, achieving greater biomass is a primary objective; however, high nutritional quality is equally important as it enhances the profitability of both forage production and the livestock enterprises it supports (Zhang et al., 2018). Crude protein is a key indicator of the nutrient availability in the forage, which is vital for feed efficiency and development of livestock. Fiber content is important for maintaining proper digestive health in ruminants. It aids in the fermentation process within the rumen, promoting a healthy microbial population and nutrient absorption (Gonzalez et al., 2020). RFV combines both ADF and NDF to provide a comprehensive measure of forage digestibility. CP content, fiber content, and RFV are the essential quality metrics used for assessing forage nutritive values (Agnew et al., 2022). These metrics not only guide the selection of appropriate fertilizer but also enhance the overall efficiency of livestock production systems, contributing to sustainable agricultural practices (Sharma et al., 2018).

The above indicators showed a marked difference between heading and maturity stages in our study, the CP and fiber content in the heading stage was greater than that in maturity stage. This observed trends in crop might be related to plant canopy characteristics and the reduction in leaf area associated with the of leaf senescence (Liu et al., 2021). Photosynthesis is the physiological base of crop growth and crop nutrient formation, while more than 90% of crop biomass is derived from photosynthetic products (Zhang et al., 2025b), and the reduction of photosynthesis in the maturity stage greatly decrease the oat nutritional quality. Moreover, the increase of stem rigidity in maturity stage led to the enhanced accumulation of fiber content (Zhang et al., 2018). These changes collectively contributed to a decline of nutritive quality in the maturity stage.

In general, increasing N/P levels and sowing density are believed to increase crop quality (Li et al., 2023; Shao et al., 2024). However, the increased N/P levels or sowing density couldn’t increase oat quality indefinitely in our study, and excess fertilization and density (N2P2 treatment and H treatment) show a detrimental effect on nutrient quality of oat. These results partly support our hypothesis that the farmers’ practiced N/P application and sowing density in the Loess Plateau of northwest China are excessive, and would limit the water and nutrient demands required for optimal growth and productivity of oat. In our study, M-N1P2 markedly improved the nutritive quality both in heading and maturity stages, evidenced by greater CP and RFV and lower NDF content compared to that of other treatment. Consistently, previous studies have also reported high forage quality of crops with optimized fertilization and sowing density (Li et al., 2021). The response of forage quality to increasing fertilization amounts and planting density follows a parabolic curvilinear relationship. Excessive fertilization and planting density frequently lead to a reduction in forage quality. This is attributed to their impact on enhancing cell wall components and fiber content (Kamran et al., 2023). These findings were corroborated by the elevated levels of NDF and ADF observed in our study. In addition, overapplication of fertilizers has been shown to reduce the protein content in grains and forages, as well as the bioavailability of essential nutrients. This suggests that there is an optimal N and phosphorus application that maximizes nutritional quality without compromising yield (Zhang et al., 2017a). Optimal N and P treatment increase crops N and P uptake and use, thereby enhancing the synthesis of amino acid and protein contents (Kaplan et al., 2019).

Moreover, our study highlights the interaction between optimizing sowing density and fertilizer rate is crucial. The combination of the optimal agronomic practices directly influences the crop’s ability to use fertilizers effectively, particularly in fertility-poor soils (Gregoire et al., 2022). The moderate sowing density of forage crops can enhance the uptake efficiency of fertilizers which significantly improve nutritive efficiency of oat forage. It is also essential for meeting the growing forage demands sustainably.

### 4.2 Oat forage yield, CP yield and N/P uptake response to fertilization and density

The forage yield response is an important variable for evaluating nutrient efficiency in agroecosystems, because it reflects the condition of nutrients in agroecosystems (Mueller et al., 2012). CP yield and N/P uptake are also important indicators directly act to reflect the overall plant nutrition and yield. The forage yield, CP yield and N/P uptake of oats exhibited a consistent trend in both years, peaked at the M-N1P2 treatment, and showed a strong correlation between CP yield, N/P accumulation and forage yield (**Figure 9**).

Some study showed that high N/P amounts are regarded as the main way to increase forage yield and productivity (Ahmad et al., 2023; Chen et al., 2024). However, excessive fertilization (N2P2) failure to produce positive outcomes for crop yield and nutrient in harvested oat forage in the present study. A study showed that under the high N and P supply, only 15% of fertilizers could transform into forage and grain and the remaining nutrients were lost as gaseous emissions or leached from the soil (Shi et al., 2016). Farmers of our experimental region apply excessive fertilizer lavishly to try to maximize crop yields, this leads to the decline of fertilizer efficiency, and crops cannot take up excess fertilizer after saturation of fertilizer uptake, which ultimately leads to the increase of planting costs and environmental pollution. Previous study showed that optimal fertilizer application could increase the crop yield by increasing the nutrient uptake and nutrient efficiency (Wang et al., 2023). This study confirmed this viewpoint that the forage yield, CP yield and N/P uptake increased under same sowing density with application of fertilizers. Thus, precisely formulating an optimal fertilizer rate should refer to the maximal fertilizer uptake for a certain crop.

In our two consecutive years of field experiments, sowing density of 150 kg ha^-1^ simultaneously have resulted in maximum forage yield, CP yield and N/P uptake, and decreasing or increasing the sowing density had negative effects on these indicators. A study indicated that the low density of oat in the fields might produce high forage yield per plant, but achieving high forage yield per hectare is difficult (Chen et al., 2024). Increase of sowing density can improve forage yield per hectare by increasing the number of plants. The increasing sowing density can also minimize the waste of applied fertilizers by increasing total tillering number and expanding root-canopy structure, thus increasing use efficiency of nutrients and improving N/P uptake and CP yield (Shao et al., 2024). However, the increased sowing density couldn’t increase oat yield indefinitely and excess population density will reduce the field yield of oat. The reason could be that too dense plant density exacerbated the competition for resources, led to excessive population density, poor ventilation, light transmission, and the breeding of diseases and pests (Zhang et al., 2021b; Hou et al., 2019), thus resulting in yield per plant loss, made difficult to achieve a high yield per hectare. Another possible reason is that the climatic and soil conditions are highly diverse in time and space (Kamran et al., 2023), and the sowing density for other regions may not be suitable for Loess Plateau, the poor soil moisture and nutrient conditions in this region do not support a larger population density of oats. Hence, a site-specific implementation strategy is needed to identify appropriate sowing density of oat in the Loess Plateau.

Notably, the oat yield was significantly influenced by the interaction between fertilization and density (**Table 1**). The positive impact of N1P2 treatment on yield was more pronounced under the M treatment compared with that under the L and H. This revealed that only an optimal sowing density can maximize the effectiveness of fertilizers, thereby significantly improving productivity and sustainability. Results underscored the importance of coupled fertilizer rate and sowing density.

### 4.3 Oat nutrient efficiency response to fertilization and density

Nutrient efficiency is an important index to evaluate forage, particularly in the context of increasing global forage demand and environmental concerns associated with nitrogen and P fertilizer use. Improving N/P efficiency can lead to enhanced forage quality and yield, which are vital for increasing productivity of livestock and agriculture. Our study showed that the N1P2 treatment significantly increased the agronomic efficiency, N fertilizer recovery efficiency and N uptake, however, the P recovery and uptake efficiency peaked at the N2P1 treatment. Thus, we used the comprehensive evaluation to calculated the comprehensive scores of N and P, and the results of evaluation showed that the N1P2 treatment still had the highest NCS and PCS. Previous study reported that the combination of N and P application not only increased grain yield but also enhanced nutrient use efficiency and agronomic efficiency in agricultural systems (Li et al., 2023). Plant N and P content and LAI increased with optimal levels of N/P application, resulting in higher nutrients use efficiency by enhancing photosynthetic rate (Nasar et al., 2022). Moreover, optimal fertilization promotes the growth of crop roots, and a vigorous root system enable crops to absorb more nutrients of soil, contributing to the higher nutrient efficiency and productivity.

Optimizing sowing density can significantly influence the root distribution, nutrient uptake efficiency and overall yield of crops. For instance, a study on winter wheat indicated that improved sowing density led to enhanced root length density and nitrogen uptake efficiency, which ultimately resulted in higher yields (Wang et al., 2012). Similarly, another investigation of maize production demonstrated that adjusting planting density not only improved the leaf area index and radiation use efficiency but also optimized N use efficiency, thereby stabilized the yield in loess plateau (Jia et al., 2018). It was consistent with our results that optimizing sowing density (M treatment) significantly increased agronomic efficiency of fertilizers and efficiency of N and P.

Moreover, a study highlighted that the application of fertilizers in conjunction with optimized planting density resulted in increased nitrogen accumulation and translocation during the critical post-anthesis phase, leading to improved yield and nitrogen use efficiency (Yuan et al., 2024). In our study, the interaction between fertilization and sowing density played a vital role in maximizing nutrients use efficiency. This synergistic approach underscores the importance of integrating agronomic practices to enhance both resource use efficiency and crop productivity, the combination of optimal sowing density and fertilization could be an excellent strategy to optimize resource use. This integrated approach not only supports sustainable agriculture but also laying the foundation for achieving high oat yield in Loess Plateau.

### 4.4 Implications for fertilizer rate and sowing density

Yield and quality indicators are important for forages, but the nutrients use efficiency is equally important, especially to avoid the waste of fertilizers. Thus, when formulating an agronomic practice, these two points should be fully balanced. Previous studies have reported a trade-off between maximizing forage productivity and nutrients use efficiency (Kamran et al., 2022; Kaplan et al., 2019). This trade-off requires both consideration of maximizing the economic benefits and minimizing environmental pollution, while formulating density and fertilization management practices.

Low nutrient use efficiency is mainly caused by unreasonable fertilizer application, mass nutrient loss, and an imbalance between crop nutrient supply and demand (Yang et al., 2016). This is evident in agricultural systems where overuse of fertilizers without considering the specific needs of the crops, particularly nitrogen and phosphorus, can result in nutrient leaching, soil acidification, and a decline in soil biodiversity. In addition to these ecological concerns, the financial implications of excessive fertilizer use and improper sowing density cannot be overlooked, the costs associated with purchasing fertilizers and seeds can escalate quickly, particularly when they lead to diminished soil health and reduced crop yields over time. This indicates that finding the optimal sowing density and fertilizer rate is essential for maintaining soil health and ecological balance.

A study has shown that optimizing sowing density and fertilization can enhance soil fertility and crop productivity while reducing the reliance on fertilizers inputs. In the semi-arid areas, reducing fertilizers application while optimizing planting density has been proved to improve yield and nitrogen use efficiency, thereby minimizing the negative impacts associated with excess fertilizers use (Wu et al., 2024). Similarly, our study indicated that the yield, quality, N use efficiency and agronomic efficiency of oats under the M-N1P2 treatment were higher than other treatments. These results revealed that the optimal density and fertilization could ensure oat yield while improving nutrients use efficiency, it showed a reduced need for fertilizers, thus reducing the negative impact of excess fertilizers input on the environment. The current study has the potential for improving productivity and promoting ecological conservation, and could be a promising strategy for the efficient production of oats in Loess Plateau.

## 5 Conclusion

The findings of the two-year field study revealed that the optimal sowing density and fertilizer rate (M-N1P2) greatly increased the CP content, relative feed value, forage yield, CP yield, agronomic efficiency, N/P content, N/P uptake, N uptake efficiency and N recover efficiency, while decreased the NDF of oat. The results of correlation analysis showed significant positive correlations of forage CP content and yield with agronomic efficiency, N/P uptake efficiency and N/P recover efficiency, but negative correlations with ADF and NDF. The results of comprehensive evaluation exhibited that M-N1P2 had the highest N comprehensive score and P comprehensive score among all treatments. Hence, the M-N1P2 treatment was able to maintain yield and quality while maximizing agronomic efficiency and N efficiency without much reduction in the P efficiency across the two growing seasons. Therefore, a sowing density of 150 kg ha^-1^ coupled with 50 kg ha^-1^ N and 90 kg ha^-1^ P is recommended as the optimal tillage practice for oats in the Loess Plateau.

Present findings illustrated significant prospects for optimizing the fertilizer rate and sowing density for oat in the Loess Plateau of northwest China. Nevertheless, the precipitation levels and soil characteristics, may vary between different semi-arid regions, and the optimal fertilizer rate and sowing density identified in this study may not be applicable for achieving maximum productivity in other semi-arid areas. Therefore, future multi-locational studies are suggested to clarify how climatic variations in different semi-arid regions would influence the potential effects of fertilization and sowing density on oat production, forage nutritive quality, and resource use efficiency. More precise guidelines will aid farmers in minimizing fertilizer and seed inputs while maximizing economic profitability within agricultural production systems.

## Data Availability

The raw data supporting the conclusions of this article will be made available by the authors, without undue reservation.
