# Supplementary material for: Optimal fertilizer rate and sowing density can improve oat quality, yield and N and P comprehensive efficiency in the Loess Plateau of China
Source: Front Plant Sci. 2025 Jun 13;16:1604427. doi: 10.3389/fpls.2025.1604427 (PMC12202502; doi:10.3389/fpls.2025.1604427)
Supplement: Supplementary file 1 [file Table1.docx]

**Table S1**

Two-way analysis of variance (ANOVA) for the effects of sowing density (SD) and fertilizer rate (FR) on crude protein (CP), acid detergent fiber (ADF) and neutral detergent fiber (NDF) of oat.

| Year | Treatment | Heading stage | | |  | Maturity stage | | |
| --- | --- | --- | --- | --- | --- | --- | --- | --- |
|  |  | CP | ADF | NDF |  | CP | ADF | NDF |
| 2022 | SD | * | * | ** |  | NS | * | ** |
|  | FR | ** | * | * |  | * | * | * |
|  | SD*FR | NS | NS | NS |  | NS | NS | NS |
|  |  |  |  |  |  |  |  |  |
| 2023 | SD | NS | * | * |  | NS | * | * |
|  | FR | ** | NS | * |  | ** | * | * |
|  | SD*FR | NS | NS | NS |  | NS | NS | NS |

Note: ** indicates significance at *P* < 0.05; * indicates significance at *P* < 0.05; ns indicates no significance.

**Table S2**

Two-way analysis of variance (ANOVA) for the effects of sowing density (SD) and fertilizer rate (FR) on nitrogen (N) content and phosphorus (P) content of oat.

| Year | Treatment | Heading stage | |  | Maturity stage | |  |
| --- | --- | --- | --- | --- | --- | --- | --- |
|  |  | N content | P content |  | N content | P content | |
| 2022 | SD | NS | NS |  | NS | NS | |
|  | FR | * | * |  | * | * | |
|  | SD*FR | NS | NS |  | NS | NS | |
|  |  |  |  |  |  |  | |
| 2023 | SD | * | * |  | * | * | |
|  | FR | ** | * |  | * | * | |
|  | SD*FR | NS | NS |  | NS | NS | |

Note: ** indicates significance at *P* < 0.05; * indicates significance at *P* < 0.05; ns indicates no significance.
